# Cytokine-Activated Mesenchymal-Stem-Cell-Derived Extracellular Matrix Facilitates Cartilage Repair by Enhancing Chondrocyte Homeostasis and Chondrogenesis of Recruited Stem Cells

**DOI:** 10.34133/research.0700

**Published:** 2025-05-21

**Authors:** Qiming Pang, Zhuolin Chen, Xinhang Li, Jingdi Zhan, Wei Huang, Yiting Lei, Wei Bao

**Affiliations:** ^1^ Department of Orthopedics, Affiliated Banan Hospital of Chongqing Medical University, Chongqing, China.; ^2^ Department of Orthopaedic Surgery, The First Affiliated Hospital of Chongqing Medical University, Chongqing, China.; ^3^ Chongqing Municipal Health Commission Key Laboratory of Musculoskeletal Regeneration and Translational Medicine, The First Affiliated Hospital of Chongqing Medical University, Chongqing, China.; ^4^ Orthopaedic Research Laboratory of Chongqing Medical University, Chongqing Medical University, Chongqing, China.; ^5^ Department of Biomedical Engineering, The Chinese University of Hong Kong, Hong Kong SAR, China.

## Abstract

Current strategies for cartilage repair, including decellularized cartilage matrices and synthetic bioactive materials, often encounter challenges such as immune responses and donor morbidity. In this study, we optimized an extracellular matrix (ECM) derived from mesenchymal stem cells through preconditioning with disease-associated inflammatory factors, specifically interleukin 6, tumor necrosis factor alpha, and interferon gamma (IFN-γ). Our in vitro experiments demonstrated that the cytokine-preconditioned stem-cell-derived ECM, especially IFN-γ-ECM, supports chondrocyte homeostasis by restoring mitochondrial energy metabolism. Furthermore, bioactive molecules secreted from this preconditioned ECM boost the recruitment of endogenous stem cells and facilitate their differentiation into chondrocytes. Notably, we found that IFN-γ-ECM facilitates the chondrogenic differentiation of mesenchymal stem cells through the activation of the integrin/phosphatidylinositol 3-kinase/Akt pathway and the Smad2/3 signaling cascade. These results highlight the potential of the cytokine-stimulated ECM, especially IFN-γ-ECM, to restore chondrocyte homeostasis, optimize the mobilization of endogenous stem cells, and substantially improve the regeneration of cartilage defects, offering a promising strategy for acellular cartilage graft reconstruction.

## Introduction

Cartilage defects frequently result from traumatic injuries, severe infections, or degenerative joint diseases, presenting substantial clinical challenges owing to cartilage’s lack of direct blood supply and the restricted availability of essential nutrients from the adjacent synovial fluid [1–4]. These injuries often progress to osteoarthritis (OA), marked by joint deformity and functional impairment [5–8]. Chondrocytes, the only type of cell found in cartilage, synthesize and secrete essential extracellular matrix (ECM) components, including type II collagen (col II), sox9, and aggrecan, essential for preserving the structure of cartilage [9,10]. However, chondrocytes exhibit mitochondrial dysfunction following injury, characterized by reduced mitochondrial numbers and diminished expression of electron transport chain proteins, leading to insufficient adenosine triphosphate (ATP) production [11]. This disruption compromises cellular bioenergetic homeostasis, resulting in chondrocyte apoptosis, decreased matrix synthesis, and increased matrix degradation [11,12]. Therefore, restoring mitochondrial function in chondrocytes is critical for initiating cartilage repair and regeneration.

Despite various treatment strategies—such as microfracture (MF), arthroscopic debridement, and cell-based therapies utilizing autologous chondrocytes or bone-marrow-derived mesenchymal stem cells (BMSCs)—these methods encounter substantial challenges, including limited availability of cells, risks of immune rejection, donor site morbidity, and logistical issues related to cell transport [13–16]. Emerging evidence indicates that diverse cell types, including synovial stem cells and BMSCs, can migrate to cartilage injury sites and enhance tissue regeneration in response to appropriate biochemical signals [17,18]. Techniques such as drilling and MF have been implemented to improve cell recruitment, although the success rates of these interventions vary [19]. To address these challenges, researchers are investigating synthetic materials, particularly polymers, to finely tune the release of growth factors and cytokines. This approach aims to enhance cell recruitment and facilitate host remodeling to promote tissue regeneration [20,21]. Nonetheless, challenges persist, as few synthetic materials exhibit sufficient bioactivity to effectively support cartilage repair [20,22]. Thus, the development of bioactive materials capable of modulating chondrocyte homeostasis while recruiting endogenous stem cells presents an encouraging method for cartilage tissue reconstruction.

In recent years, the quest to overcome the limitations of natural cartilage for osteochondral tissue engineering has sparked interest in engineered ECMs (eECMs) derived from mesenchymal stem cells (MSCs) [23]. Traditional approaches, leveraging lyophilized or decellularized cartilage, have demonstrated potential due to their inherent ability to induce cartilage formation [24,25]. Decellularized cartilage sheets (acellular cartilage sheets) have emerged as promising bioscaffolds for cartilage regeneration when combined with chondrocytes or BMSCs [26]. However, the deterioration rate and incorporation challenges within acellular cartilage sheet layer sandwich structures pose substantial obstacles [27]. Additionally, the dense, nonporous nature of a natural cartilage ECM hampers the mobilization and migration of endogenous stem cells, limiting its efficacy in situ [28]. In contrast, an MSC-derived ECM offers a promising alternative due to its unique composition and inherent structural properties, which furnish a supportive microenvironment conducive to cell homing, proliferation, and differentiation [29,30]. Notably, these characteristics have been shown to enhance tissue repair capabilities [29]. For example, Liu et al. [31] established that osteogenically differentiated MSC-derived ECM scaffolds significantly improved bone repair in a rat cranial defect model. This enhancement occurred by promoting MSC proliferation and osteogenic differentiation, despite the lack of an osteogenic inducer like dexamethasone, and facilitated mineralization similar to that of bone tissue.

The traditional method of generating an ECM usually involves plating MSCs onto cell culture plates and incubating them in serum-supplemented media for 1 to 2 weeks. However, the ECM produced using this conventional method often fails to satisfy the varied requirements needed for effective tissue engineering applications [32]. As a result, optimizing the culture conditions has emerged as a crucial strategy to refine and improve the functionality and performance of the ECM. Common pre-treatment strategies include adding specific components to the culture medium and altering serum concentrations or oxygen levels during incubation. However, these strategies are often nonspecific and may not provide optimal therapeutic effects for specific diseases [32]. To tackle these challenges, we previously utilized Luminex liquid-phase microarray technology to evaluate the production of inflammatory mediators in the joint fluids of OA patients compared to that in healthy controls. This analysis identified significant differences in the concentrations of interleukin 6 (IL-6), tumor necrosis factor alpha (TNF-α), and interferon gamma (IFN-γ) among nearly 30 inflammatory factors [33]. These cytokines are crucial in the pathogenesis of OA and cartilage matrix degeneration, and they may activate MSCs to enhance ECM components for cartilage repair [34–37]. Therefore, we selected these 3 cytokines for MSC preconditioning in this study, aiming to improve the efficacy and bioactivity of the resulting ECM and provide a robust foundation for constructing a functional MSC-derived ECM that addresses specific regenerative challenges.

**Fig. 1.**
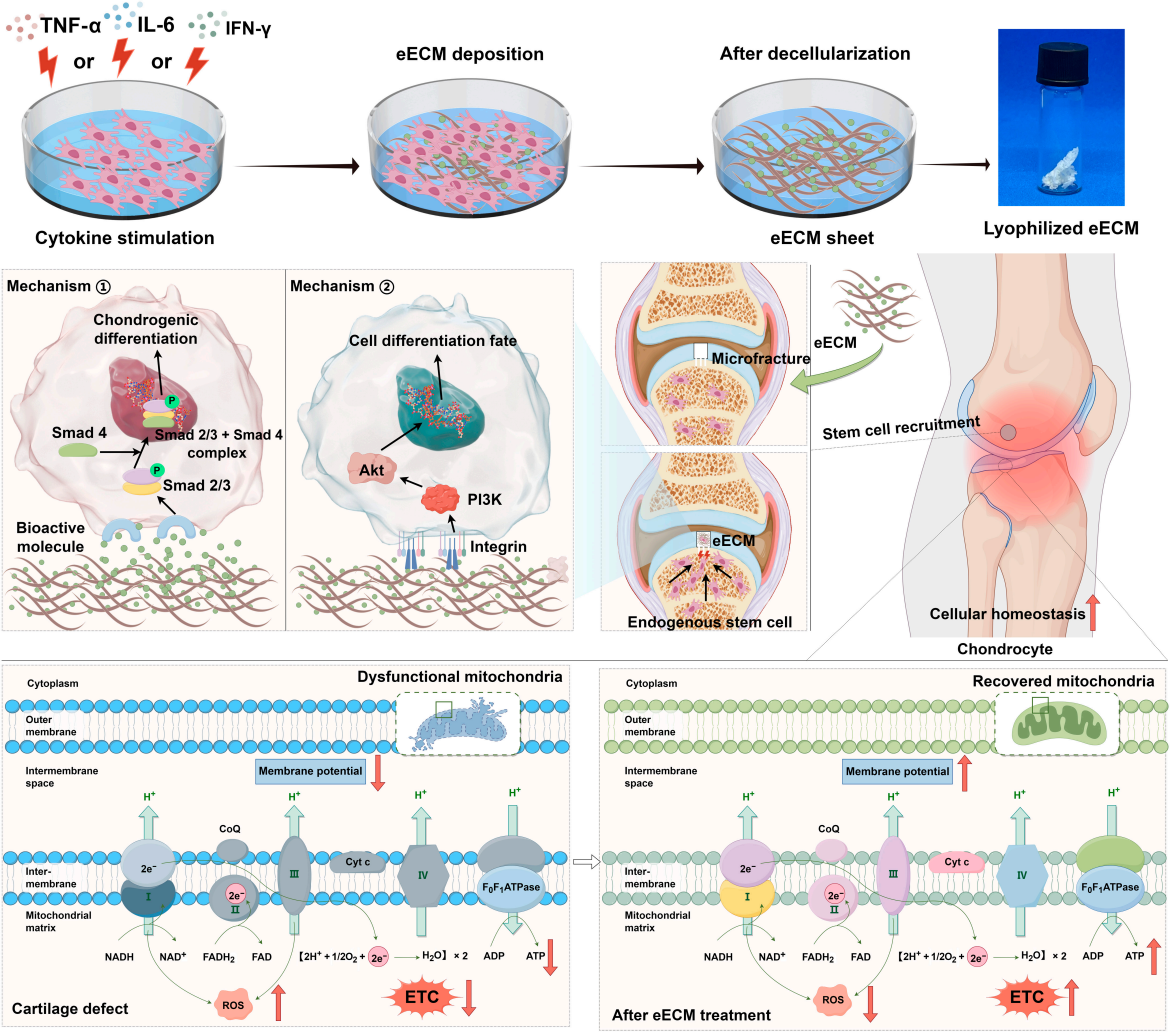
Illustration of the engineered extracellular matrix (eECM) enhancing cartilage repair. The eECM improves chondrocyte function and energy metabolism and promotes mesenchymal stem cell (MSC) recruitment and differentiation via transforming growth factor beta (TGF-β)/Smads and integrin/phosphatidylinositol 3-kinase (PI3K)/Akt pathways. It is applied to cartilage defects, leading to regeneration. TNF-α, tumor necrosis factor alpha; IL-6, interleukin 6; IFN-γ, interferon gamma; CoQ‌, ubiquinone; Cyt c‌, cytochrome c; ‌NADH‌, reduced nicotinamide adenine dinucleotide; NAD‌, nicotinamide adenine dinucleotide; FADH_2_, reduced flavin adenine dinucleotide; FAD‌, flavin adenine dinucleotide; ADP, adenosine diphosphate; ATP, adenosine triphosphate; ROS, reactive oxygen species; ETC, electron transport chain.

Taken together, we sought to engineer an MSC-derived ECM that enhances chondrocyte function and facilitates the recruitment of endogenous stem cells. This was achieved through a strategic approach combining cytokine preconditioning with in vitro culture techniques. By leveraging MSCs’ ability to secrete abundant ECM proteins and bioactive molecules during culture, we sought to create a matrix that mimics the dynamic characteristics of native cartilage. Specifically, we pre-treated MSCs with the inflammatory mediator IL-6, TNF-α, or IFN-γ to improve the quality and bioactivity of the ECM produced. Through comparative analysis, we aim to determine the cytokine preconditioning regimens that best support chondrocyte homeostasis and the mobilization of stem cells to cartilage injury sites. Furthermore, we explored how these specific conditions regulate chondrocyte function and support the differentiation of the migrating stem cells toward a chondrogenic lineage. Ultimately, we intend to transplant the most effective MSC–ECM constructs in vivo to evaluate their regenerative capabilities in cartilage defects (Fig. 1). Our findings will advance the understanding of how eECMs can improve cartilage repair, offering insights into new therapeutic methods for OA and other cartilage-associated disorders.

## Results and Discussion

### Fabrication and assessment of the MSC-derived ECM

**Fig. 2.**
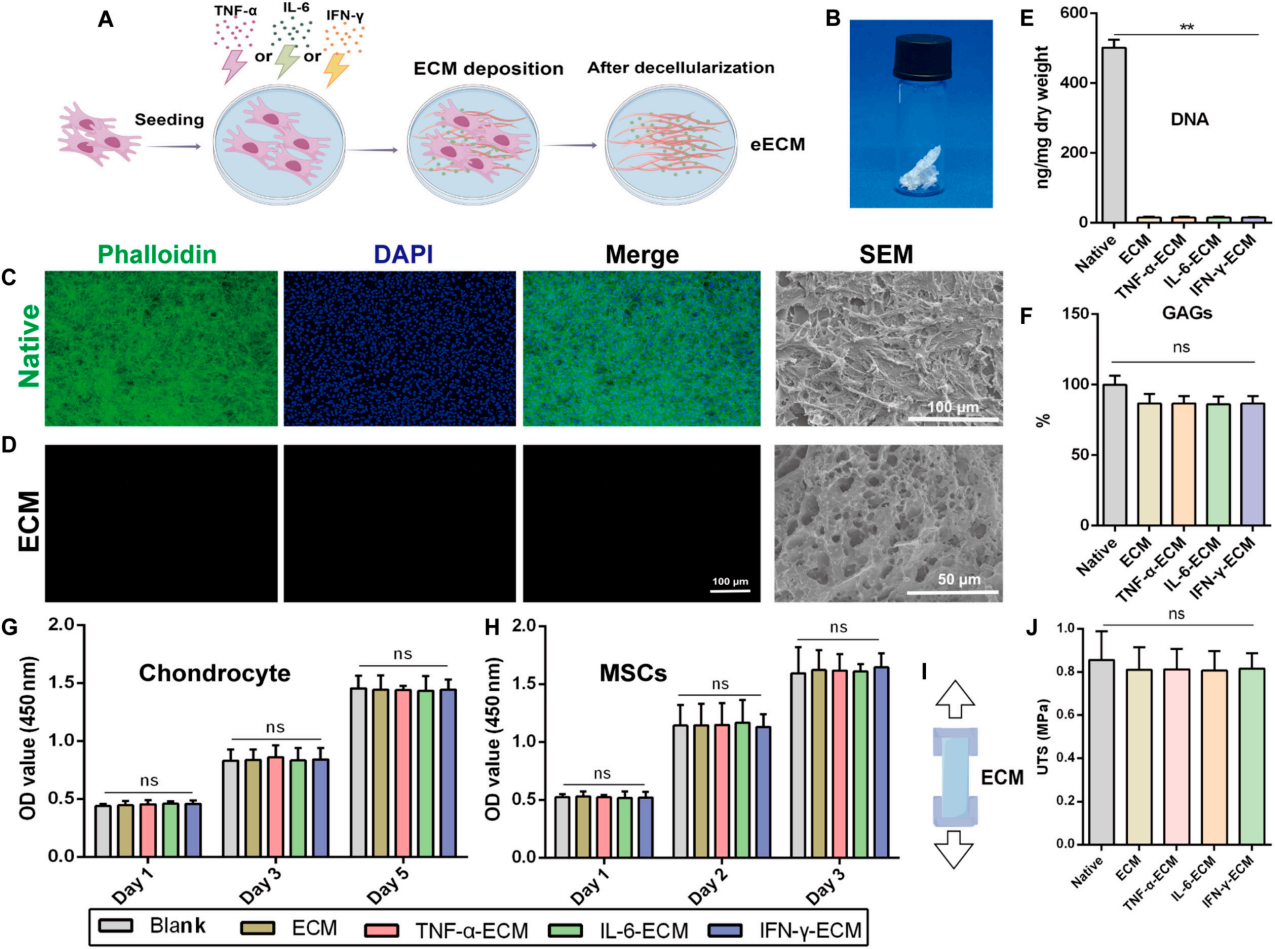
Preparation and characterization of an engineered ECM. (A) Diagram illustrating the generation of an engineered extracellular matrix (ECM) from MSCs exposed to various inflammatory cytokines. (B) Lyophilized ECM sheets following decellularization. (C) Immunofluorescence (IF) and scanning electron microscopy (SEM) images of MSCs prior to decellularization. (D) IF and SEM images of MSCs postdecellularization. (E) DNA content in ECMs before and after decellularization. (F) Glycosaminoglycan (GAG) content in ECMs before and after decellularization. (G) Cell Counting Kit-8 (CCK-8) assay results evaluating chondrocyte viability after exposure to engineered ECMs on days 1, 3, and 5. (H) CCK-8 assay results assessing MSC viability after exposure to engineered ECMs on days 1, 2, and 3. (I) Ultimate tensile strength (UTS) testing pattern diagram. (J) Comparison of UTSs across different groups. *P < 0.05; **P < 0.01; ns, not significant; *n* = 3. DAPI, 4′,6-diamidino-2-phenylindole.

Decellularization techniques have proven to be promising methods for generating bioactive ECM scaffolds [38]. These techniques successfully eliminate cellular components while maintaining the protein structure and biochemical constituents of the ECM, thereby maintaining intrinsic cues critical for cell recruitment and tissue regeneration [36]. To assess the impact of inflammatory factors on an MSC-derived ECM, we cultured MSCs in the context of IL-6, TNF-α, or IFN-γ for 2 weeks. Following cytokine stimulation, we applied a previously established decellularization protocol to generate a biologically active eECM (Fig. 2A).

Immunofluorescence (IF) staining revealed intact cytoskeletal structures and nuclei in MSC sheets prior to decellularization, which were markedly absent postdecellularization (Fig. 2C and D). Scanning electron microscopy images displayed distinct stacks of growing cells before decellularization (Fig. 2C), while the lyophilized ECM appeared as a translucent white membrane (Fig. 2B), revealing a network of interconnected pore-like structures where the cellular architecture had been lost (Fig. 2D). Quantitative analysis indicated that the DNA content significantly decreased from 501.81 ± 18.38 ng/mg in natural MSCs to less than 20 ng/mg after decellularization, well below the threshold of 50 ng/mg (Fig. 2E). In contrast, the glycosaminoglycan (GAG) content remained stable throughout the process (Fig. 2F). The ultimate tensile strength (UTS) analysis, illustrated in Fig. 2I, revealed no significant differences between groups pre- and postdecellularization (Fig. 2J), suggesting that our freeze–thaw method effectively preserved essential ECM components, including collagen and GAGs.

To evaluate the impact of degradation and release products from the eECM on cultured chondrocytes and MSCs, we performed a Cell Counting Kit-8 (CCK-8) assay. Our results demonstrated continued cell proliferation without statistical differences across groups (Fig. 2G and H), indicating that the eECM possesses excellent biocompatibility. These findings suggest that the eECM demonstrates strong biocompatibility and functions as an effective cellular scaffold, offering a robust 3-dimensional environment that supports the growth of endogenous chondrocytes and stem cells.

### Proteomic analysis of the eECM

**Fig. 3.**
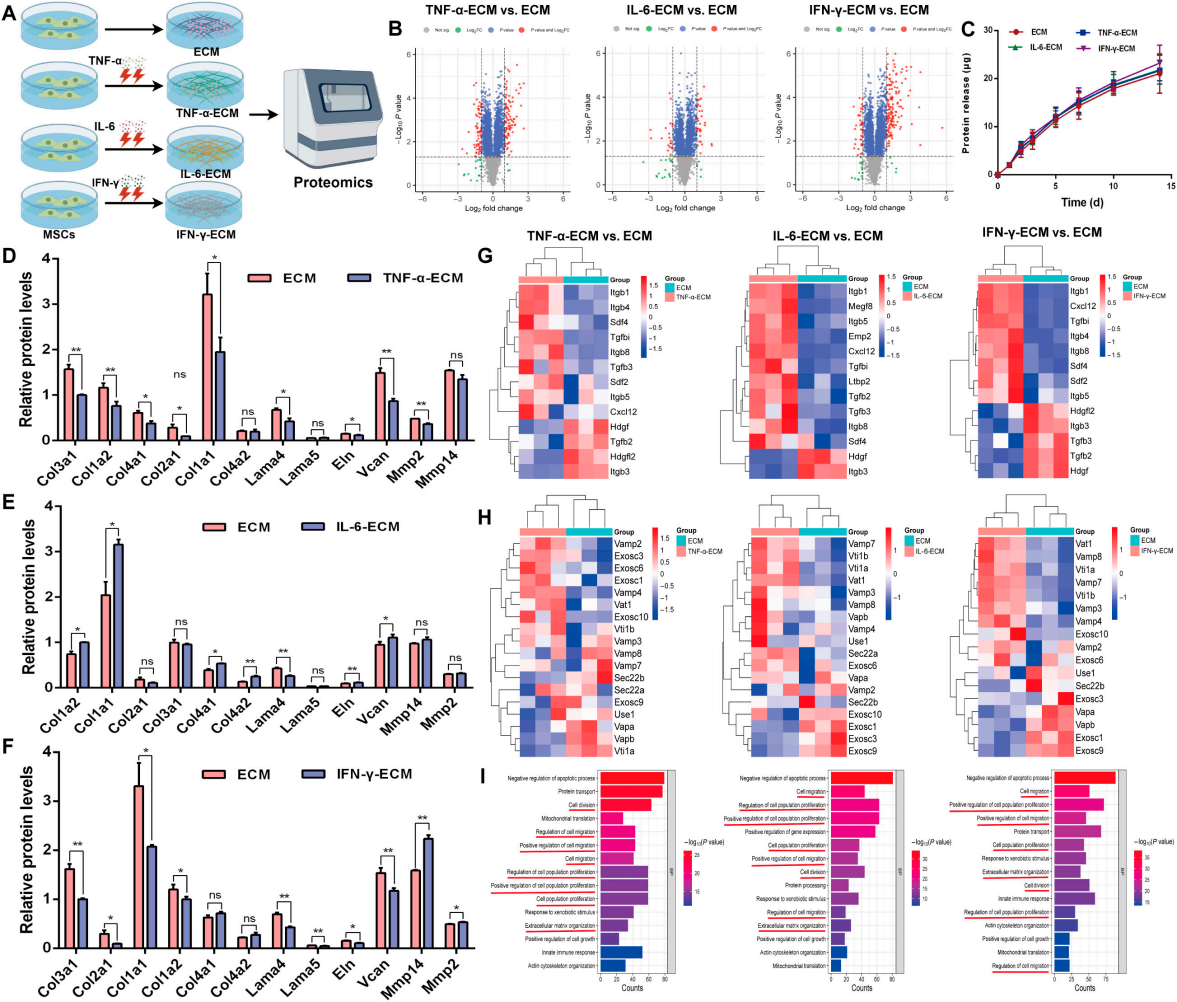
Proteomic analysis revealing extensive alterations in the ECM induced by inflammatory cytokine stimulation. (A) Diagram summarizing the proteomic profiles of ECMs derived from MSCs with and without inflammatory cytokine treatment (*n* = 3). (B) Volcano plots displaying differential protein expression between the ECM and engineered ECMs. (C) Protein release profiles from engineered ECMs quantified using the bicinchoninic acid (BCA) method. (D to F) Effects of inflammatory cytokine stimulation on key ECM components. *P < 0.05; **P < 0.01. (G) Identification of differences in bioactive molecules between the ECM and engineered ECMs through proteomic analysis. (H) Profiling of proteins associated with extracellular vesicles and exosomes within the ECM. (I) Gene Ontology (GO) enrichment analysis of differentially expressed proteins comparing the ECM to engineered ECMs. Eln, elastin; Vcan, versican; BP, biological process.

To better understand the characteristics of the eECM, we performed a comprehensive proteomic characterization (Fig. 3A). The results revealed significant alterations in protein composition following exposure to inflammatory cytokines. Specifically, the ECM derived from TNF-α-stimulated MSCs exhibited an up-regulation of 1,010 differential proteins and a down-regulation of 1,068 proteins compared to the unmodified ECM. In parallel, the ECMs from IL-6- and IFN-γ-stimulated MSCs showed changes of 813 and 859 up-regulated proteins and 847 and 935 down-regulated proteins, respectively (Fig. 3B). Notably, analysis of key ECM components, including collagen, laminin (Lama), matrix metalloproteinases (MMPs), elastin (Eln), and versican (Vcan) (Fig. 3D to F), indicated that cytokine stimulation substantially modulated their expression levels.

The paracrine signaling capabilities of MSCs, which involve the secretion of different bioactive molecules, including cytokines along with growth factors, have gained substantial attention in tissue engineering [39–41]. The ECM functions not only as a structural scaffold for cells but also as a reservoir for cytokines and signaling molecules that regulate cellular functions [36,42]. Among the differentially expressed proteins, bioactive factors like TGFBI, TGFB3, TGFB2, SDF2, SDF4, and CXCL12 were identified (Fig. 3G), playing critical roles in chondrocyte recruitment, stem cell chondrogenic differentiation, and maintenance of chondrocyte homeostasis [43–45]. Extracellular vesicles (EVs), facilitated by adhesion molecules and integrins, serve as key mediators of intercellular communication, transferring various biomolecules impacting physiological and pathological processes [46,47]. Proteins linked to EVs and exosome synthesis, including VAMP3, VAMP4, VAMP7, and exosome complex components (EXOSC1, EXOSC3, EXOSC6, EXOSC9, and EXOSC10), were detected in our ECM samples (Fig. 3H). Exosome markers CD9, CD63, and CD81, typical of MSC-derived exosomes, were also found in both the ECM and eECM (Fig. S1, Supplementary Materials). This suggests the potential presence of EVs or exosomes in the MSC-derived ECM, which may be essential for maintaining chondrocyte homeostasis and facilitating the chondrogenic differentiation of stem cells. Kinetic studies of soluble molecule release from both the ECM and eECM demonstrated a sustained release profile, creating a favorable microenvironment for chondrogenesis (Fig. 3C). Analysis of Gene Ontology (GO) enrichment for the differentially expressed proteins showed that the eECM was significantly enriched for processes related to cell migration, positive regulation of cell proliferation, and ECM organization (Fig. 3I). These findings underscore the considerable potential of the eECM to support chondrocyte homeostasis and enhance tissue regeneration.

### Effect of the eECM on chondrogenic matrix biosynthesis in chondrocytes

The overproduction of pro-inflammatory cytokines, coupled with abnormal mechanical stress, inflicts substantial damage on chondrocytes, disrupting the delicate balance between cartilage ECM synthesis and degradation [48,49]. This imbalance is further exacerbated by the degradation of col II and sox9, both crucial components synthesized by chondrocytes, through enzymes such as ADAMTS5 (a disintegrin and metalloproteinase with thrombospondin motifs 5) and MMP13 [50]. In this research, we explored the potential of the cytokine-preconditioned stem-cell-derived ECM to counteract these deleterious effects by modulating anabolic and catabolic activities within chondrocytes.

**Fig. 4.**
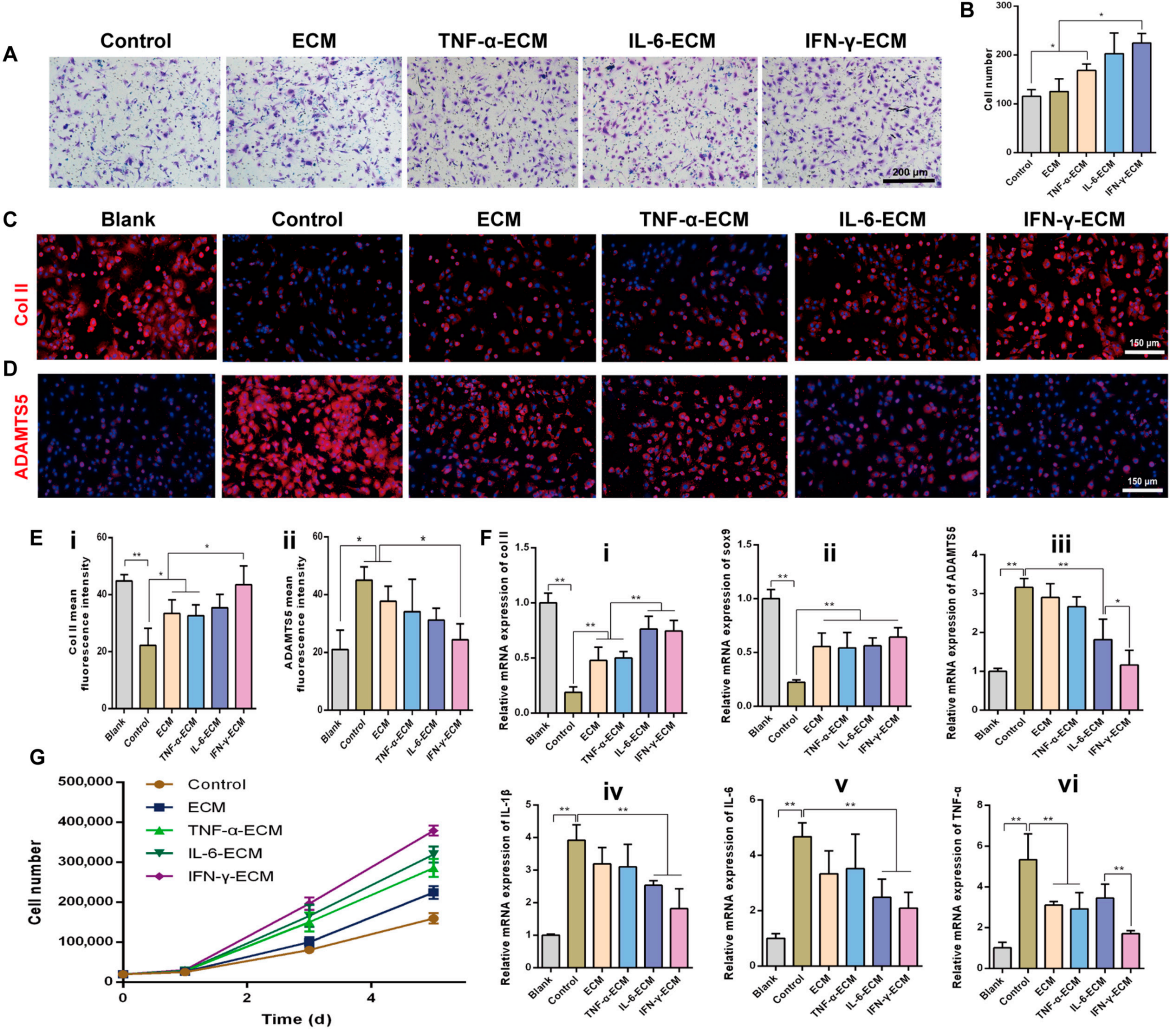
Engineered ECM modulates chondrocyte homeostasis. (A) Impact of engineered ECMs on the migration of chondrocytes. (B) Quantification of migrated chondrocytes. (C) IF staining showing type II collagen (col II) synthesis in interleukin 1β (IL-1β)-induced chondrocytes administered with engineered ECMs. (D) IF staining revealing ADAMTS5 (a disintegrin and metalloproteinase with thrombospondin motifs 5) synthesis in IL-1β-induced chondrocytes administered with engineered ECMs. (E) Quantitative analysis of fluorescence intensity for col II (i) and ADAMTS5 (ii). (F) Reverse transcription quantitative real-time polymerase chain reaction (RT-qPCR) results showing gene expression for col II (i), sox9 (ii), ADAMTS5 (iii), IL-1β (iv), IL-6 (v), and TNF-α (vi). (G) Assessment of the effects of engineered ECMs on the proliferation of IL-1β-induced chondrocytes. *P < 0.05; **P < 0.01. mRNA, messenger RNA.

Our results demonstrate that the eECM profoundly influences the expression of critical cartilage matrix elements. IF analysis revealed that chondrocytes exposed to 20 ng/ml of interleukin 1β (IL-1β) for 1 d exhibited a substantial decline in col II and an up-regulation of ADAMTS5 compared to untreated controls. Remarkably, treatment with eECMs, particularly IFN-γ-ECM, resulted in a marked restoration of col II levels and a decrease in ADAMTS5 expression, indicating its potent anabolic effect (Fig. 4C to E). The reverse transcription quantitative real-time polymerase chain reaction (RT-qPCR) data corroborated these results, showing enhanced expression levels of col II and sox9 in chondrocytes treated with eECMs. Notably, IFN-γ-ECM and IL-6-ECM significantly reduced ADAMTS5 expression, suggesting a robust suppression of catabolic activities (Fig. 4F). These findings suggest that IFN-γ-ECM is particularly effective in restoring cartilage homeostasis by promoting ECM synthesis and inhibiting its degradation.

Beyond the anabolic–catabolic balance, eECMs also exhibited significant effects on chondrocyte recruitment and proliferation. Chemotaxis assays indicated a marked rise in chondrocyte migration, with IFN-γ-ECM demonstrating the strongest recruitment capability (Fig. 4A and B), crucial for facilitating endogenous cartilage repair. The study also tackled chondrocyte senescence, characterized by reduced proliferation and the senescence-associated secretory phenotype, which increases cytokine secretion [51,52]. RT-qPCR showed increased cytokine expression in chondrocytes stimulated by IL-1β, but IL-6-ECM and IFN-γ-ECM treatment substantially lowered levels of TNF-α, IL-6, and IL-1β, with IFN-γ-ECM exhibiting the strongest anti-inflammatory effect (Fig. 4F). Cell counting and CCK-8 assays revealed that the eECMs, particularly IFN-γ-ECM, significantly enhanced chondrocyte proliferation on days 3 and 5, suggesting a reversal of senescence and restored proliferative capacity in IL-1β-induced chondrocytes (Fig. 4G and Fig. S2, Supplementary Materials).

We acknowledge that our use of healthy murine chondrocytes exposed to acute IL-1β does not fully replicate the chronic nature of human OA. However, our findings offer valuable insights into the therapeutic potential of eECMs, particularly IFN-γ-ECM, in modulating the cartilage matrix environment. This approach not only enhances chondrocyte recruitment and proliferation but also balances anabolic and catabolic activities, highlighting the need for future studies to incorporate longer-term models and human tissues to further validate these insights.

### Effects of the eECM on energy metabolism in chondrocytes

**Fig. 5.**
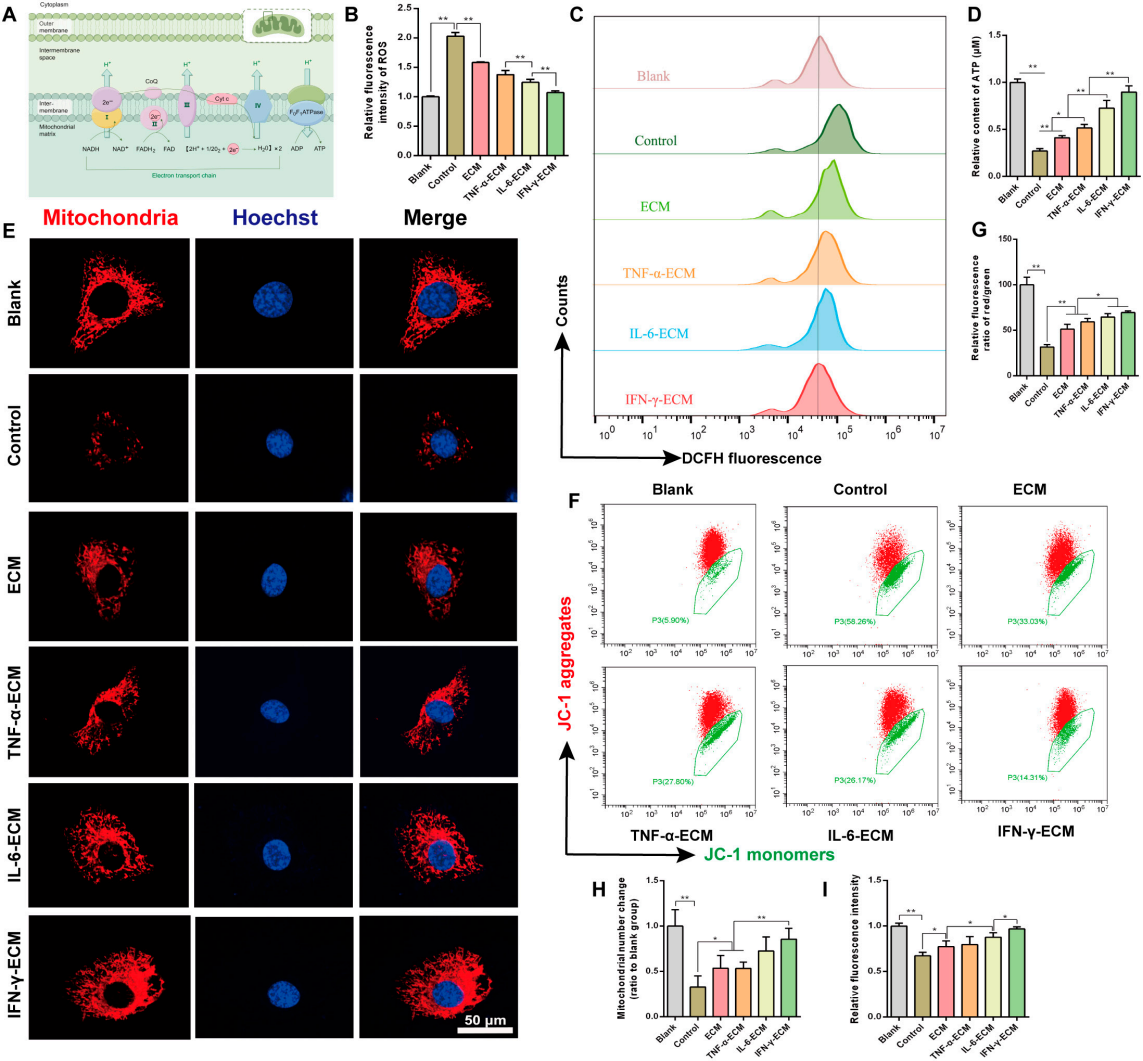
Impact of engineered ECMs on mitochondrial energy metabolism. (A) Schematic illustrating oxidative phosphorylation within the mitochondrial inner membrane. (B and C) Flow cytometry (FCM) assessment of cellular ROS levels using 2′,7′-dichlorodihydrofluorescein diacetate (DCFH-DA) staining. (D) Quantification of ATP levels in each experimental group. (E) IF images showing mitochondrial morphology. (F and G) Analysis of mitochondrial membrane potential through JC-1 staining. (H) Quantification of the mitochondrial fluorescence intensity from panel (E). (I) Quantification of the mitochondrial count shown in panel (E). *P < 0.05; **P < 0.01.

Mitochondria, as illustrated in Fig. 5A, represent crucial organelles enclosed by membranes consisting of inner and outer layers. The inner mitochondrial membrane hosts vital protein complexes engaged in oxidative reactions within the respiratory chain, alongside ATP synthase (complex V), predominantly responsible for converting adenosine diphosphate to ATP in the substrate [49,53]. The electron transport chain is widely recognized as a primary generator of reactive oxygen species (ROS). While healthy cells regulate ROS levels to support metabolic functions and maintain homeostasis, overproduction of ROS can compromise mitochondrial performance and disturb cellular metabolism. A comparative analysis between normal chondrocytes and those affected by OA reveals a reduction in the dynamics of respiratory chain complexes in OA chondrocytes. This decline leads to oxidative stress, an increased release of inflammatory mediators, accelerated matrix degradation, and heightened rates of apoptosis and cellular senescence [49,54,55]. Therefore, restoring mitochondrial functionality and metabolic processes in OA chondrocytes is vital for maintaining cellular homeostasis.

Analysis of reactome enrichment regarding the differentially expressed proteins showed significant pathways associated with metabolism, protein metabolism, and lipid metabolism (Fig. S3, Supplementary Materials). This indicates that the eECM could significantly restore mitochondrial energy metabolism. In order to analyze the impact on energy metabolism in IL-1β-induced chondrocytes, we first assessed intracellular ROS levels utilizing the 2′,7′-dichlorodihydrofluorescein diacetate (DCFH-DA) probe. Flow cytometry results, illustrated in Fig. 5B and C, indicated a marked decrease in ROS levels after eECM treatment, with the most significant reduction in the IFN-γ-ECM group (Fig. 5B and C and Fig. S4, Supplementary Materials). ATP levels, assessed using the ATP Assay Kit, demonstrated increased ATP production in IL-1β-induced chondrocytes treated with eECMs compared to the control group, with no notable difference between the IFN-γ-ECM and blank groups (Fig. 5D). Considering the vital role of aerobic respiration in chondrocyte energy metabolism, we assessed mitochondrial quantity and membrane potential (ΔΨm) across treatment groups. Confocal laser scanning microscopy of stained mitochondria revealed significant increases in both mitochondrial count and membrane potential following eECM treatment (Fig. 5E, H, and I). These findings were corroborated by flow cytometry, showing an increase in green fluorescence (indicating lowered ΔΨm) and a decline in red fluorescence (indicating elevated ΔΨm) in chondrocytes. eECM treatment notably enhanced the ratio of red to green fluorescence, indicating restored mitochondrial membrane potential (Fig. 5F and G). These findings suggest that eECMs, particularly IFN-γ-ECM, can enhance mitochondrial electron transport chain function and activity in IL-1β-induced chondrocytes, improving cellular energy metabolism.

### The influence of the eECM on stem cell migration and recruitment

Recent research has revealed the role of bone marrow as a rich source of resident stem cells for cartilage repair [19]. MF techniques are frequently employed to stimulate the recruitment of these stem and progenitor cells from the bone marrow cavity for cartilage regeneration [56]. However, research has shown that the number of endogenous repair cells available after MF procedures is often insufficient, primarily due to restricted migration and localized retention [57,58]. Therefore, there is a critical need to design a cell-free scaffold with optimized structural and functional properties, combined with MF, to improve the recruitment of endogenous cells and support effective cartilage regeneration.

**Fig. 6.**
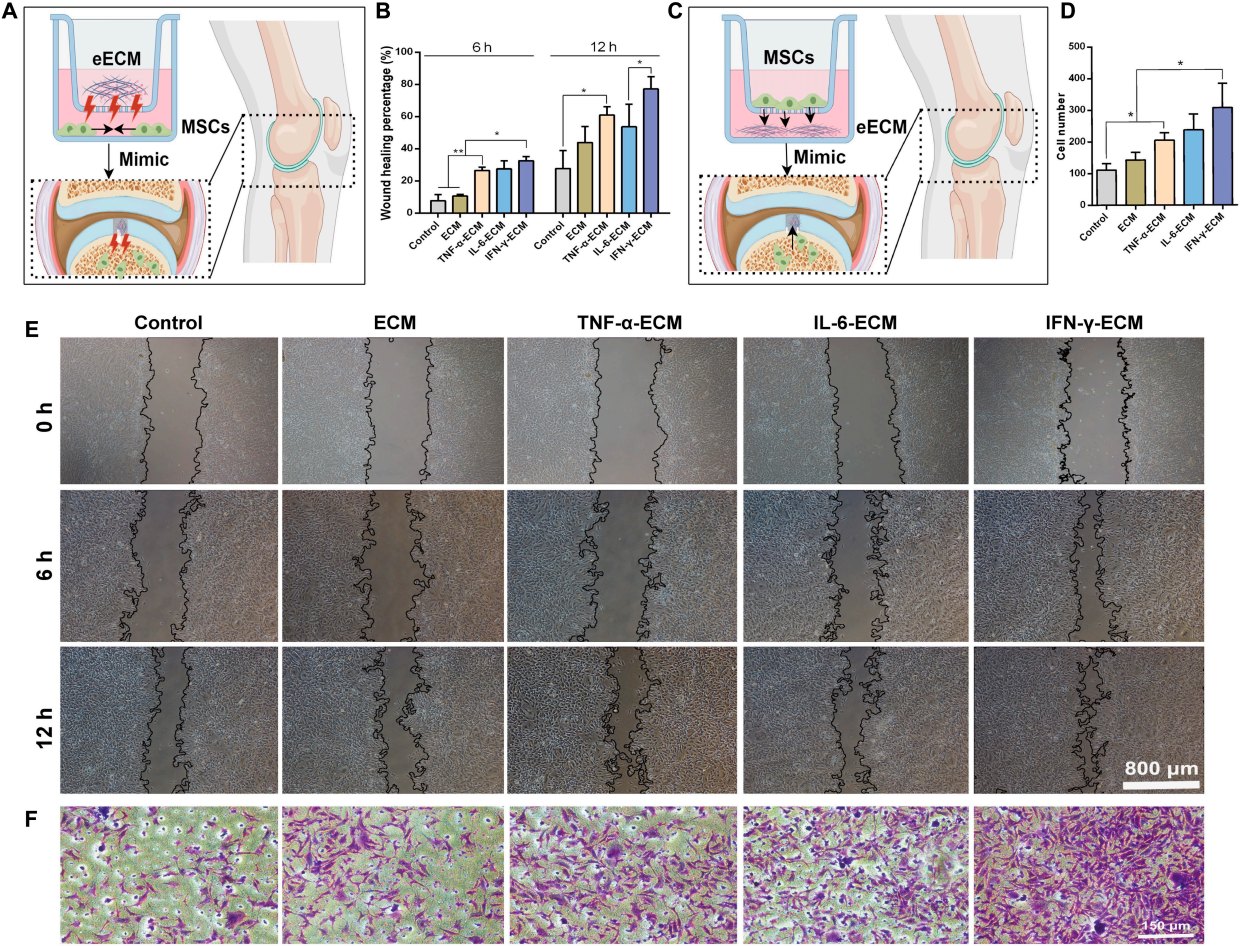
Impact of engineered ECMs on MSC migration. (A) Schematic overview of the scratch wound healing assay. (B) Graph depicting the percentage of wound closure over time after treatment with engineered ECMs. (C) Schematic overview of the chemotaxis assay. (D) Results showing the number of migrated MSCs in response to engineered ECMs. (E) Scratch assay evaluating the influence of engineered ECMs on MSC migration. (F) Migration assay assessing the recruitment of MSCs in response to engineered ECMs. *P < 0.05; **P < 0.01.

To determine the recruitment capacity of our eECM, we performed a scratch assay using C3H10T1/2 cells seeded in the lower chamber and the eECM positioned in the upper chamber (Fig. 6A). Bioactive molecule release from the ECM facilitated migration toward the scratch area. Notably, the IFN-γ-ECM group showed significantly enhanced wound closure compared to both the control and standard ECM groups, indicating its superior ability to promote cellular migration (Fig. 6B and E). Furthermore, we utilized a Transwell chemotaxis assay to further investigate the migratory behavior of C3H10T1/2 cells in response to the eECM. In this configuration, the eECM was positioned in the lower compartment, while C3H10T1/2 cells were added to the upper compartment (Fig. 6C). The findings revealed a substantial rise in the number of cells migrating into the lower compartment containing the eECM, with the IFN-γ-ECM group exhibiting the most pronounced effect (Fig. 6D and F). This indicates that the eECM effectively stimulates endogenous stem cells to disengage from their ecological niches and migrate into the articular cavity, likely mediated through the controlled release of CXCL12 and other bioactive molecules.

### Indirect regulation of stem cell chondrogenesis by the eECM

To evaluate the in vitro chondrogenic capacity of the eECM, we developed an indirect co-culture system using Transwell plates. In this configuration, the eECM was positioned in the upper chamber, while C3H10T1/2 cells were cultured in the lower chamber. This setup enabled the gradual secretion of bioactive compounds from the ECM to stimulate chondrogenic differentiation of the cells. Critical markers of hyaline cartilage, including GAGs and col II, were used as key indicators of effective chondrogenic induction.

**Fig. 7.**
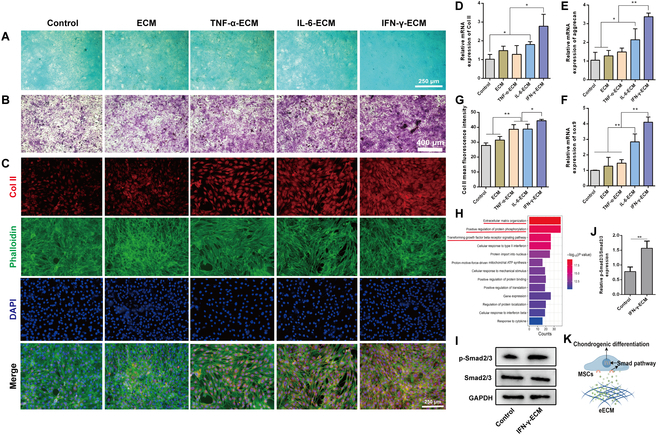
Indirect co-culture of engineered ECMs and MSCs enhances chondrogenic differentiation. (A) Alcian Blue staining images after a fortnight of chondrogenic culture. (B) Toluidine blue staining images after a fortnight of chondrogenic culture. (C) IF analysis showing the impact of engineered ECMs on col II synthesis in MSCs. (D to F) RT-qPCR results for gene expression levels of col II (D), aggrecan (E), and sox9 (F). (G) Quantification of col II fluorescence intensity. (H) GO enrichment analysis comparing differentially expressed proteins in native vs. engineered ECMs. (I) Western blot analysis for expression of phosphorylated Smad2/3 (p-Smad2/3) and total Smad2/3 proteins. (J) Quantitative evaluation of the p-Smad2/3 to total Smad2/3 protein ratio. (K) Schematic illustrating bioactive molecules from engineered ECMs that promote chondrogenic differentiation through the Smad signaling pathway. GAPDH, glyceraldehyde-3-phosphate. *P < 0.05; **P < 0.01.

The secretion of cartilage ECM was evaluated using Alcian Blue and toluidine blue staining. The staining intensity in the eECM groups, especially in the IFN-γ-ECM group, was markedly greater than that in the control group (Fig. 7A and B), suggesting that the eECM effectively stimulates the secretion of cartilaginous ECM components from C3H10T1/2 cells via the gradual release of bioactive molecules. To further validate the chondrogenic potential of the eECM, we analyzed the expression of col II using IF. The results indicated no significant differences in col II expression levels between the TNF-α-ECM and IL-6-ECM groups; however, both groups demonstrated significantly higher expression compared to the control. Notably, col II expression in these groups remained lower than that in the IFN-γ-ECM group, underscoring the superior ability of IFN-γ-ECM to promote the secretion of chondrogenic markers (Fig. 7C and G). RT-qPCR further supported these findings, demonstrating that the gene expressions of col II, sox9, and aggrecan were markedly increased in the IL-6-ECM group compared to those in the control, standard ECM, and TNF-α-ECM groups, although still lower than those in the IFN-γ-ECM group (Fig. 7D to F). Together, these results strongly suggest that the eECM, particularly IFN-γ-ECM, markedly enhances the expression of chondrogenesis-related genes, highlighting its potential for cartilage repair applications.

To better understand the mechanisms involved in the chondrogenic response, we conducted a GO enrichment analysis of differentially expressed proteins between the IFN-γ-ECM and control ECM groups. The analysis highlighted significant enrichment in processes including ECM organization, positive regulation of protein phosphorylation, and the transforming growth factor beta receptor signaling pathway (Fig. 7H). Prior research has established that activation of the canonical transforming growth factor beta/Smad signaling pathway plays a pivotal role in driving chondrogenic differentiation in stem cells [59]. Supporting this, Western blot analysis uncovered that IFN-γ-ECM significantly promoted the levels of phosphorylated Smad2/3 (p-Smad2/3) compared to the control group (Fig. 7I to K).

In summary, our results demonstrate that the eECM, especially IFN-γ-ECM, exhibits robust chondrogenic induction potential. Its capacity to enhance the differentiation of endogenous stem cells into chondrocytes offers a promising approach for cartilage repair and regeneration.

### Direct regulation of stem cell chondrogenesis by the eECM

**Fig. 8.**
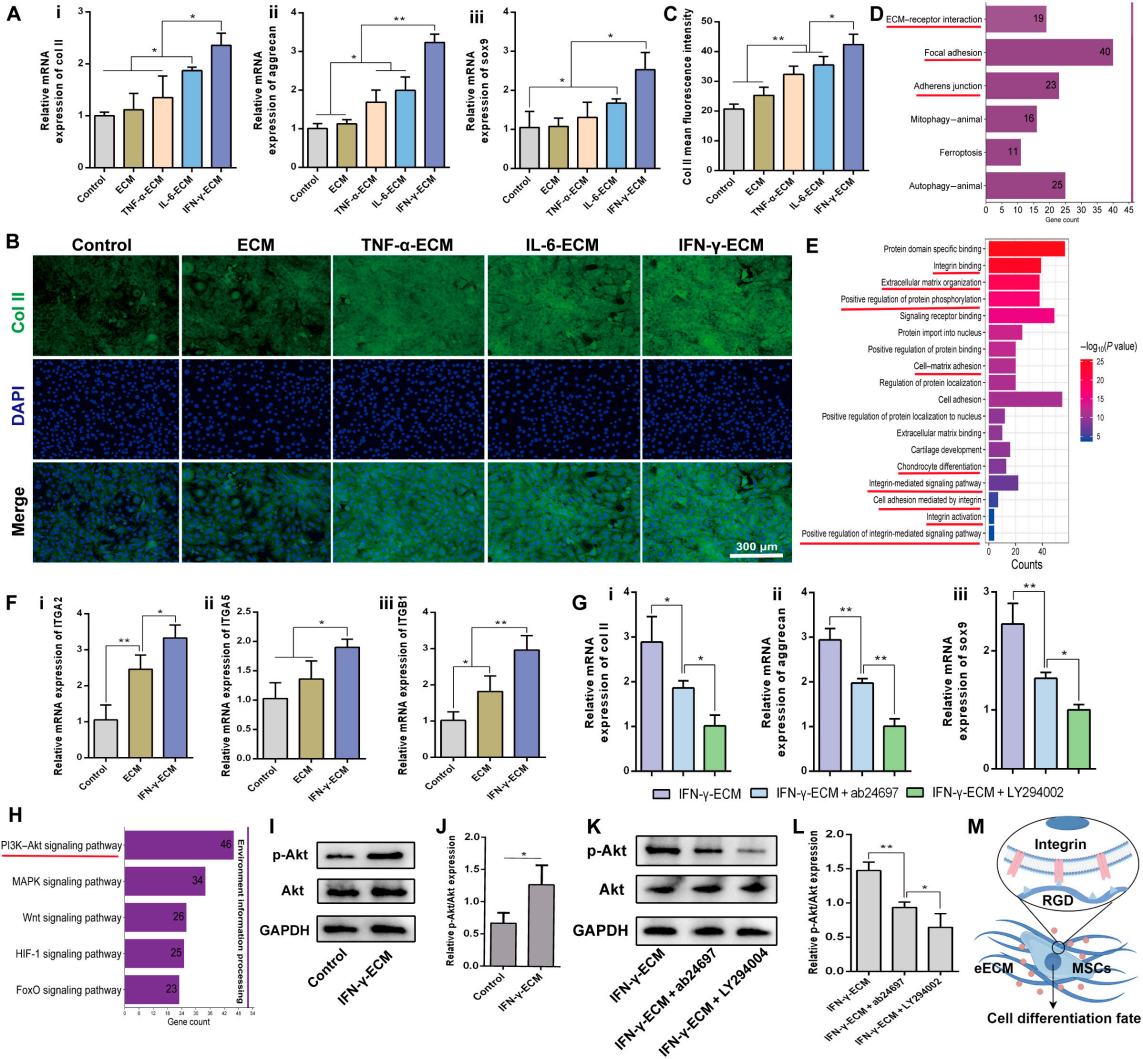
Impact of engineered ECMs on the chondrogenic differentiation of MSCs through direct co-culture. (A) RT-qPCR analysis of the intracellular expression of col II (i), aggrecan (ii), and sox9 (iii) in MSCs cultured with engineered ECMs. (B) Immunofluorescence imaging revealing the synthesis of col II in MSCs influenced by the engineered ECM. (C) Quantitative evaluation of col II fluorescence intensity in MSCs. (D) Kyoto Encyclopedia of Genes and Genomes (KEGG) pathway enrichment analysis of differentially expressed proteins comparing the ECM and IFN-γ-ECM. (E) GO enrichment analysis of differential proteins associated with the ECM and IFN-γ-ECM. (F) RT-qPCR assessment of the gene expression levels of integrin subunits ITGA2 (i), ITGA5 (ii), and ITGB1 (iii) in MSCs. (G) RT-qPCR analysis of the gene expression for col II (i), aggrecan (ii), and sox9 (iii) in MSCs. (H) KEGG analysis highlighting the number of differentially expressed proteins in pathways related to environmental information processing between the ECM and IFN-γ-ECM. (I and K) Western blot analysis exhibiting phosphorylated Akt (p-Akt) and total Akt protein expressions in MSCs. (J and L) Quantitative assessment of the ratio of p-Akt to total Akt protein levels in MSCs. (M) Schematic representation of the chondrogenic differentiation process of MSCs mediated by integrin binding to the engineered ECM. *P < 0.05; **P < 0.01. RGD, Arg-Gly-Asp.

To further explore the chondrogenic capability of the eECM, we developed a direct co-culture system in which C3H10T1/2 cells were cultured in direct contact with the ECM to evaluate the impact of bioactive sequences present on the ECM surface. RT-qPCR analysis demonstrated that the gene expressions of col II, sox9, and aggrecan in the IL-6-ECM group were significantly elevated compared to those in the control, standard ECM, and TNF-α-ECM groups, although still lower than those in the IFN-γ-ECM group (Fig. 8A). These results were additionally validated by IF assays (Fig. 8B and C), confirming that the eECM, particularly IFN-γ-ECM, effectively enhances the expression of cartilage ECM-associated genes and proteins, primarily through the bioactive sequences embedded within the ECM.

To further elucidate the underlying mechanisms, we conducted Kyoto Encyclopedia of Genes and Genomes pathway enrichment analysis of differentially expressed proteins between the IFN-γ-ECM and control ECM groups. The analysis identified significant enrichment in pathways associated with ECM–receptor interactions, focal adhesion, and adherens junctions (Fig. 8D). Additionally, GO enrichment analysis indicated substantial involvement of integrin-related pathways, including integrin binding, integrin-mediated signaling, and cell adhesion processes mediated by integrins (Fig. 8E). These findings suggest that C3H10T1/2 cells may recognize the bioactive sequences of IFN-γ-ECM through integrins on their surface, subsequently activating intracellular signaling cascades that regulate cartilage-associated pathways.

Further analysis of specific integrin subunits demonstrated that the expressions of ITGA2, ITGA5, and ITGB1—genes encoding essential integrin proteins—were greatly higher in the IFN-γ-ECM group than in both the control and standard ECM groups, as assessed by RT-qPCR (Fig. 8F). The enrichment analysis also highlighted that the phosphatidylinositol 3-kinase (PI3K)/Akt pathway was notably enriched among the differentially expressed proteins, indicating a important role in environmental signal processing during chondrogenic differentiation (Fig. 8H).

To examine whether integrins mediate chondrogenic differentiation through the PI3K/Akt pathway, we performed inhibition experiments using integrin-neutralizing antibodies (ab24697) and the PI3K inhibitor LY294002. Results showed that the expression of col II, sox9, and aggrecan in the IFN-γ-ECM group was greatly diminished following treatment with ab24697. Further reduction of these markers was observed after treatment with LY294002, indicating a critical role for the PI3K/Akt pathway in mediating chondrogenesis (Fig. 8G). Correspondingly, Western blot analysis confirmed that the treatment with IFN-γ-ECM significantly up-regulated phosphorylated Akt (p-Akt) levels, which were reduced upon inhibition with ab24697 and further diminished with LY294002 treatment (Fig. 8I to L).

These findings collectively indicate that IFN-γ-ECM promotes chondrogenesis not only by activating the integrin/PI3K/Akt signaling pathway but also potentially through additional Akt-mediated mechanisms to enhance chondrogenic differentiation. The ability of IFN-γ-ECM to robustly modulate integrin signaling and downstream effects highlights its potential as a therapeutic tool in cartilage repair strategies (Fig. 8M), offering new insights into the utilization of bioactive ECM modifications for enhanced tissue engineering applications.

### The role of the eECM in treating cartilage defects

**Fig. 9.**
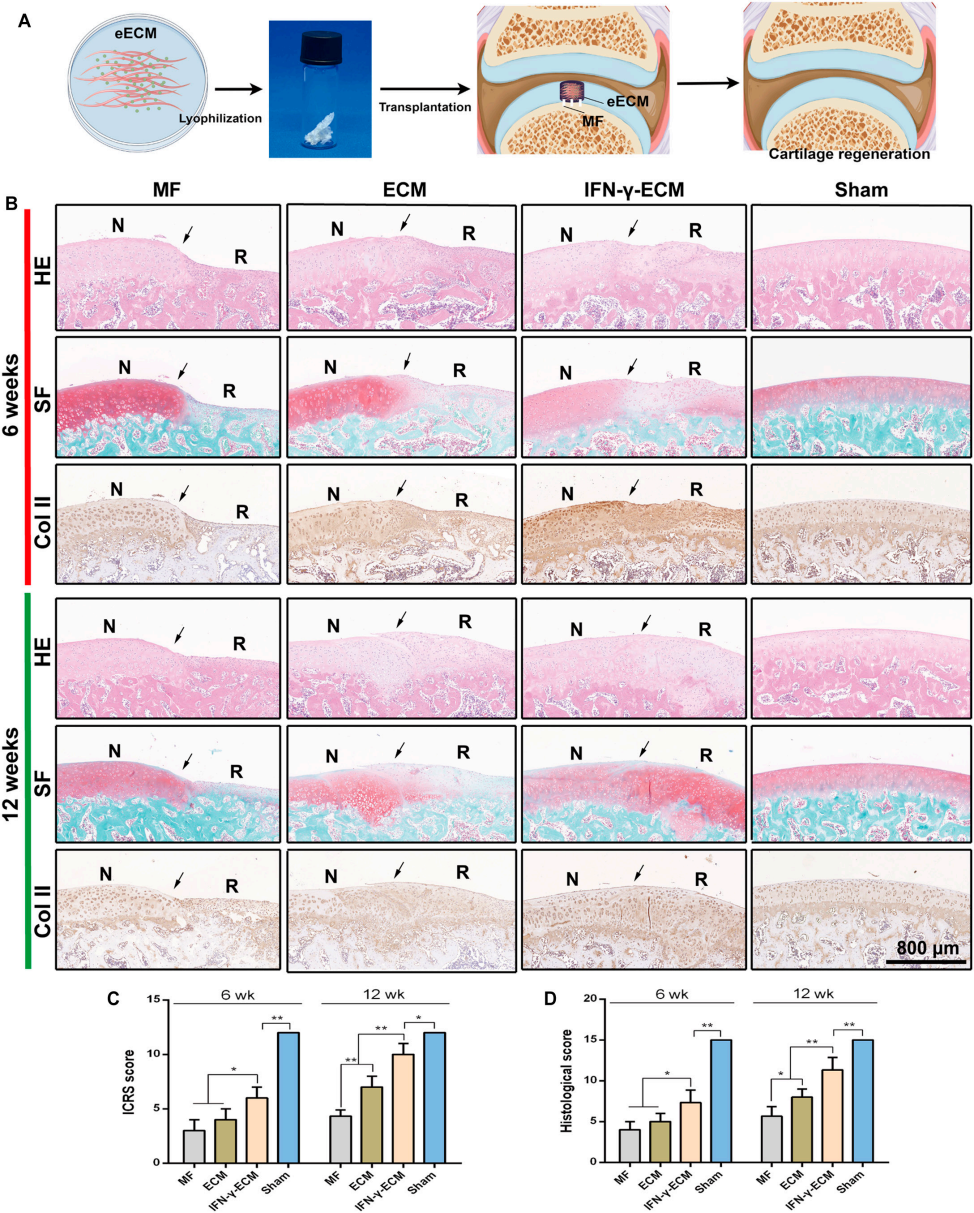
Histological assessment of repaired tissue across treatment groups at 6 and 12 weeks postsurgery. (A) Schematic representation of eECM grafting applied to cartilage defects. (B) Representative images of hematoxylin and eosin (HE), Safranin-O/Fast Green (SF), and col II immunohistochemical staining. Arrows denote the margins of the defects; N indicates normal cartilage and R represents repaired cartilage. (C) International Cartilage Repair Society (ICRS) scores of repaired tissues in each group at 6 and 12 weeks postsurgery. (D) Histological scores of repaired tissues across groups at 6 and 12 weeks postsurgery. MF, microfracture. *P < 0.05; **P < 0.01.

Based on our eECM studies, which demonstrate the ability to regulate chondrocyte homeostasis and enhance chondrogenic differentiation of stem cells, we identified IFN-γ-ECM as a promising candidate for in vivo grafting to treat cartilage defects. The MF technique is widely employed for cartilage repair as it facilitates bone marrow blood effusion from the subchondral bone, forming a clot that aids tissue healing by initiating cell homing and migration through hemostatic signals [60]. However, the endogenous repair cell level post-MF surgery often falls short due to insufficient migration and local cell retention [57,58]. To address these limitations, we combined MF with IFN-γ-ECM transplantation, hypothesizing that IFN-γ-ECM would not only sustain chondrocyte homeostasis but also recruit and direct endogenous stem cell differentiation toward cartilage to repair defects. Upon implantation into the defect site, ECM scaffolds integrated rapidly with blood; the quick coagulation created a composite material that completely filled the defect (Fig. 9A).

Histological analyses, including hematoxylin and eosin (HE) staining, Safranin-O/Fast Green (SF) staining, and col II immunostaining, revealed significantly enhanced cartilage regeneration in the IFN-γ-ECM group compared to that in the control group. At 6 weeks postimplantation, significant tissue indentation was evident in both the MF and ECM groups, with clear demarcations between neoplastic and surrounding normal tissues. In contrast, IFN-γ-ECM scaffolds supported superior hyaline cartilage regeneration, marked by the absence of obvious borders at defect sites. By week 12, the distinction between new and native tissue was still prominent in the MF and ECM groups; however, in the IFN-γ-ECM group, integration improved notably, diminishing these boundaries. Additionally, intense col II staining in repaired tissues highlighted the superior regenerative capacity of IFN-γ-ECM (Fig. 9B). Histological scoring indicated progressive improvement in the scaffold groups, with the International Cartilage Repair Society (ICRS) scores greatly higher in the IFN-γ-ECM group than in the MF and standard ECM groups at both 6 and 12 weeks postimplantation (Fig. 9C and D). Overall, the IFN-γ-ECM scaffold exhibited enhanced cellular filling, cartilage matrix formation, and robust col II presence, indicating its efficacy in promoting in vivo hyaline cartilage regeneration.

## Conclusion

In conclusion, our study highlights the transformative potential of MSC-derived ECM engineered through the strategic application of inflammatory cytokines, particularly IFN-γ. By optimizing the composition of these ECMs, we have demonstrated that they effectively restore mitochondrial energy metabolism in chondrocytes, thereby promoting cellular homeostasis. Furthermore, the eECM demonstrates a strong capacity to secrete bioactive molecules that not only attract endogenous stem cells but also facilitate their chondrogenic differentiation by activating key signaling pathways, including Smad2/3 and integrin/PI3K/Akt. These results highlight the utility of eECMs as a novel therapeutic strategy for enhancing cartilage regeneration, providing a viable alternative to traditional decellularized matrices and synthetic materials. The implications of this research extend beyond mere structural support; they offer marked advancements in the conceptualization of cell-free grafts for articular cartilage reconstruction. Future investigations should seek to translate these promising in vitro results into clinical applications, ultimately contributing to improved outcomes for patients suffering from cartilage defects.

## Materials and Methods

### Fabrication of the eECM

To generate an eECM from the C3H10T1/2 MSC line (Procell, China), cell cultures were sustained in alpha minimum essential medium (α-MEM; Gibco, USA) enriched with 10% fetal bovine serum and 1% penicillin/streptomycin (Beyotime, China). Following a 24-h adhesion phase, the medium was refreshed with α-MEM containing specific cytokines: 0.05 μg/ml IFN-γ, 0.02 μg/ml TNF-α, or 0.02 μg/ml IL-6 (Solarbio, China). The cells were cultured under these conditions for 2 weeks to generate distinct eECMs. For decellularization, the cells underwent 5 freeze–thaw cycles, whereby they were immersed in liquid nitrogen for 120 s and then placed in ultrapure water at 37 °C for 600 s to induce cell lysis. Following this, the ECM was treated with 15 mM NH_4_OH for 20 min with gentle agitation to eliminate any remaining cellular debris, followed by 5 rinses with ultrapure water to wash away residual chemicals. The success of the decellularization process was evaluated by extracting DNA both before and after treatment utilizing a DNA extraction kit (Tiangen, China), with concentrations subsequently measured using a spectrophotometer. Additionally, the GAG content in the ECM was measured utilizing an enzyme-linked immunosorbent assay kit (Meimian, China). For optimal storage and therapeutic use postpreparation, we recommend storing the eECM at −80 °C or in liquid nitrogen for long-term preservation. It is crucial to freeze-dry or lyophilize the ECM prior to storage to prevent moisture-related degradation. Upon retrieval, the ECM should be kept at a low temperature to avoid repeated freeze–thaw cycles before reconstitution and used shortly after reconstitution to enhance therapeutic outcomes. The native and eECM samples were prepared and sent to Shanghai OE Biotech Co., Ltd. for proteomic analysis, allowing for comprehensive characterization of how inflammatory cytokines affected the ECM components.

### Extraction and cultivation of primary chondrocytes

Primary chondrocytes were extracted from 1- to 2-week-old suckling mice in compliance with approved ethical guidelines. Following euthanasia by cervical dislocation, hind limbs were dissected, and femoral heads were collected. The cartilage tissue was carefully chopped into small pieces and treated with a collagenase type II solution (1 mg/ml, Sigma-Aldrich, USA) in phosphate-buffered saline (PBS) at 37 °C for 8 h with gentle agitation. After digestion, the mixture was passed through a 70-μm mesh filter to eliminate debris. Chondrocytes were subsequently centrifuged by centrifugation at 300 × g for 300 s, resuspended in complete culture medium, and maintained in standardized conditions (37.0 ± 0.1 °C, 5% CO_2_, 95% humidity) for further experimental use.

### Biocompatibility assessment

To assess the cytocompatibility of the eECM, we seeded chondrocytes and C3H10T1/2 cells in the lower chamber of Transwell plates (0.4-μm pores), with the eECM placed in the upper chamber. Cell growth and division was evaluated utilizing a CCK-8 assay (Beyotime, China) at specific time points: 1, 3, and 5 d for chondrocytes and 1, 2, and 3 d for C3H10T1/2 cells. For the assay, 10 μl of CCK-8 solution was precisely dispensed to each well of a 96-well plate, which was then maintained at 37 °C in the dark for 60 min. Absorbance was determined at 450 nm using a microplate reader.

### Mechanical testing

To evaluate the tensile properties of the scaffolds, tissue strips measuring 8 × 10 mm were prepared from C3H10T1/2 cell slices and decellularized ECM. The samples were straightened under an initial load of 0.005 N and secured in custom titanium clamps to ensure uniform force dispersion. To keep the samples moist during testing, PBS was sprayed on them. The strips were then subjected to tensile stress at a velocity of 2 mm/min until rupture occurred. The maximum stress at rupture was measured as the UTS, calculated using the formula *σ* = Fb/So, where Fb is the peak force at rupture and So is the pristine transverse section of the specimen. UTS values are reported in newtons per square millimeter (MPa).

### Protein release assessment

To assess protein release from the eECM, samples were cultured in single wells of a 96-well plate and immersed in 100 μl of PBS at 37 °C on a shaker platform. Supernatants were harvested at scheduled intervals, and the released proteins were quantified using Beyotime’s Enhanced Bicinchoninic Acid (BCA) Protein Assay Kit (China), allowing the measurement of cumulative protein release over the course of the study.

### Cell recruitment measurement

To examine the recruitment influence of the eECM on chondrocytes, we conducted a Transwell migration assay. Chondrocytes were cultured in the upper chamber of Transwell inserts with an 8-μm pore size (Corning, USA), while the eECM was cultured in the lower chamber. Following 24-h culture, the upper chamber was immobilized with 4% paraformaldehyde (PFA) solution. The cells were stained with 0.5% crystal violet solution for 15 min, and residual nontransmigrated cells on the upper surface were eliminated with cotton swabs. The migrated cells were subsequently washed 2 times with PBS and quantified using a light microscope.

To further examine the recruitment effect of the eECM on C3H10T1/2 cells, a parallel experiment was conducted. C3H10T1/2 cells were placed in the upper chamber of the same Transwell inserts, while the ECM constructs were placed in the lower chamber. After a 24-h incubation period, the cell samples were fixed with 4% PFA solution, dyed with crystal violet, and quantified following the same protocol as described for the chondrocyte assays, ensuring uniformity in experimental conditions.

Additionally, a cell scratch assay was performed for quantification assess of C3H10T1/2 cell immigration in the presence of the eECM. In this assay, the eECM was placed in the upper chamber of Transwell inserts with a 0.4-μm pore size, while C3H10T1/2 cells were seeded in the lower chamber. Once the cells reached confluence (70% to 80%), a scratch was made linearly across the cell monolayer using a 200-μl pipette tip that had been autoclaved. PBS was applied 3 times to the wells to wash away any remaining cellular debris, and serum-free medium was applied to minimize the impact of cell proliferation on the migration assessment. Cell migration into the scratched area was monitored at 0, 6, and 12 h, with images captured to quantify the extent of migration in response to the eECM.

### IF staining

After allowing the cells to adhere, they were immobilized with 4% PFA solution for 15 min at suitable temperature to preserve morphology and antigenicity. Subsequently, cells were permeabilized with 0.1% Triton X-100 in PBS for 600 s to enhance antibody accessibility to intracellular proteins. To reduce nonspecific binding, cells were incubated with 5% bovine serum albumin in PBS for 60 min. Primary antibodies targeting col II and ADAMTS5 (1:200, Servicebio, China) were prepared in accordance with the manufacturer’s guidelines and incubated at 4 °C for 12 to 16 h. Following a triple rinse with PBS, secondary antibodies conjugated to specific fluorophores were applied and allowed to incubate for 1 h at room temperature. Samples were then treated with phalloidin for 30 min and rinsed 3 times with PBS. Finally, nuclei were counterstained with DAPI (Servicebio, China), and the samples were subsequently measured via fluorescence microscopy.

### Reverse transcription quantitative real-time polymerase chain reaction

Total RNA was obtained utilizing an RNA isolation kit (Thermo Fisher, USA) and subsequently reverse transcribed into complementary DNA. Following complementary DNA synthesis, RT-qPCR was conducted using the SYBR Green fluorescent dye to quantify gene expression. To normalize target gene expression, glyceraldehyde-3-phosphate dehydrogenase (GAPDH) was employed as an internal control, and relative expression was calculated utilizing the 2^−ΔΔCT^ method. Table S1 provides details on the specific primers used in this research.

### Effect of the eECM on the proliferation of IL-1β-induced chondrocytes

To assess how the eECM influences IL-1β-induced chondrocyte proliferation, chondrocytes were seeded at a concentration of 20,000 cells per well. The cells were then treated with IL-1β (20 ng/ml) for 1 d. Following this initial stimulation, they were exposed to the eECM for 1, 3, and 5 d, with samples collected at every checkpoint for further analysis. Cell proliferation was assessed by examining absorbance utilizing a CCK-8 (Beyotime, China) enzyme marker at the specified intervals.

### Mitochondrial staining

Mitochondrial staining in viable chondrocytes was carried out using MitoTracker Red CMXRos (Beyotime, China) as per the manufacturer’s instructions. Briefly, upon culture medium removal, the MitoTracker Red CMXRos working solution was introduced and kept at 37 °C in a 5% CO_2_ incubator. Subsequently, the cell samples were stained with 1% Hoechst for 10 min. Imaging was performed utilizing confocal laser scanning microscopy to visualize the mitochondrial staining patterns within the chondrocytes.

### Analysis of mitochondrial membrane potential

Following treatment, the cell samples were treated with the JC-1 working solution at 37 °C in a 5% CO_2_ environment for 20 min. Postincubation, the cells underwent 2 washes with JC-1 staining buffer. Flow cytometry was employed to quantify JC-1 fluorescence, with red aggregates detected in the FL2 channel and green monomers in the FL1 channel. The resulting data were processed and analyzed using the CytExpert software (Beckman Coulter, Fullerton, USA).

### ROS detection

Following the specified treatments, chondrocytes were washed twice with PBS and treated with 10 μM DCFH-DA (Beyotime, China) for 20 min in the dark. After cultivation, the cells were rinsed with PBS to remove excess dye. Initial fluorescence imaging was conducted utilizing a fluorescence microscope to evaluate ROS expression qualitatively. For quantitative analysis, ROS fluorescence intensity was measured using flow cytometry to provide detailed and precise data.

### ATP measurement

To measure the intracellular ATP content in chondrocytes, we utilized the Enhanced ATP Assay Kit (Beyotime, China) in accordance with the manufacturer’s instructions. Briefly, chondrocytes were lysed using the kit’s lysis buffer, and the resulting lysates were centrifuged to remove debris. The clarified supernatants were combined with the ATP detection working solution, and luminescence was measured using a luminometer.

### Alcian Blue staining

Cells were cultured in 6-well plates, fixed with 4% paraformaldehyde, and cultivated with Alcian Blue solution for 30 min at room temperature. Following PBS washing, bright-field microscopy was used to capture images.

### Toluidine blue staining

The cellular toluidine blue staining protocol involves seeding cells in 6-well plates, fixing them with 4% paraformaldehyde, and subsequently incubating them with toluidine blue solution for 30 min at a suitable temperature. Following staining, cells are rinsed with PBS and visualized using bright-field microscopy.

### Western blotting

Protein samples were extracted from cell lysates, and their concentrations were quantified utilizing a BCA protein assay kit. Equal protein quantities were resolved on a 10% sodium dodecyl sulfate–polyacrylamide gel electrophoresis gel and subsequently transferred to a polyvinylidene difluoride membrane. The membrane was blocked with 5% nonfat milk to avoid nonspecific binding and then probed overnight at 4 °C with primary antibodies specific to GAPDH, p-Smad2/3, Smad2/3, p-Akt, and Akt (1:1,000, Abiowell, China). After thorough washing, horseradish peroxidase-conjugated secondary antibodies were applied to the membrane, and the protein bands were detected using an enhanced chemiluminescence detection system, and their intensities were analyzed using the ImageJ software for quantification.

### In vivo animal studies

The research was carried out in accordance with the ethical guidelines approved by the Ethics Committee of the First Affiliated Hospital of Chongqing Medical University. Twenty-four male Sprague Dawley rats, aged 8 weeks, were used in the experiments. Surgical procedures involved exposing the knee joint and creating a cartilage defect (2 mm in diameter and 1 mm in depth) in the patellar groove, followed by local MF. The rats were randomly assigned to 4 different experimental groups: one receiving only MF treatment (MF), another receiving MF along with ECM implantation (ECM), a third group receiving microfracture with IFN-γ-ECM implantation (IFN-γ-ECM), and, finally, a sham-operated group. The sham group underwent identical surgical procedures without inducing cartilage defects or MFs.

At 6 and 12 weeks postsurgery, the rats were sacrificed, and knee joints were collected. The joints were immobilized with 4% PFA solution and decalcified for 1 month before paraffin embedding. Histological examination was performed on sections using HE and SF staining to evaluate the morphology and arrangement of newly formed tissues. Additionally, immunohistochemical staining was performed to evaluate col II expression in the regenerated cartilage. Tissue regeneration was scored using the ICRS histological scoring system for a comprehensive assessment.

### Statistical analysis

All experiments were implemented with at least 3 biological replicates (*n* ≥ 3), and results are reported as mean ± standard deviation. Statistical analysis was carried out utilizing the SPSS 22 software. To compare differences across multiple groups, one-way analysis of variance was utilized. For pairwise comparisons, independent-samples *t* tests were adopted. Statistical significance was characterized as a *P* value of less than 0.05 (*P* < 0.05).

## Data Availability

The data supporting the findings of this study are available from the corresponding authors upon reasonable request.
