# Supplementary material for: Cytokine-Activated Mesenchymal-Stem-Cell-Derived Extracellular Matrix Facilitates Cartilage Repair by Enhancing Chondrocyte Homeostasis and Chondrogenesis of Recruited Stem Cells
Source: Research (Wash D C). 2025 May 21;8:0700. doi: 10.34133/research.0700 (PMC12494090; doi:10.34133/research.0700)
Supplement: Supplementary 1 — Figs. S1 to S4 Table S1 [file research.0700.f1.docx]

**Cytokine-Activated MSC-Derived ECM Facilitates Cartilage Repair by Enhancing Chondrocyte Homeostasis and Chondrogenesis of Recruited Stem Cells**

Qiming Pang^b,^^c,d1^, Zhuolin Chen^b,c,d1^, Xinhang Li^b,c,d1^, Jingdi Zhan ^b^^,c,d^, Wei Huang^b,c,d^*, Yiting Lei^b,c,d,e^**, Wei Bao ^a,b,c,d^***

^a^ Department of Orthopedics, Affiliated Banan Hospital of Chongqing Medical University, Chongqing, China

^b^ Department of Orthopaedic Surgery, The First Affiliated Hospital of Chongqing Medical University, Chongqing, China

^c^ Chongqing Municipal Health Commission Key Laboratory of Musculoskeletal Regeneration and Translational Medicine, The First Affiliated Hospital of Chongqing Medical University, Chongqing, China

^d^ Orthopaedic Research Laboratory of Chongqing Medical University, Chongqing Medical University, Chongqing, China

^e^ Department of Biomedical Engineering, The Chinese University of Hong Kong, NT, Hong Kong SAR, China

*Corresponding author: huangw511@163.com

**Corresponding author: leiyit614@163.com

***Corresponding author: 137301@hospital.cqmu.edu.cn

^1^The authors contributed equally to this work.


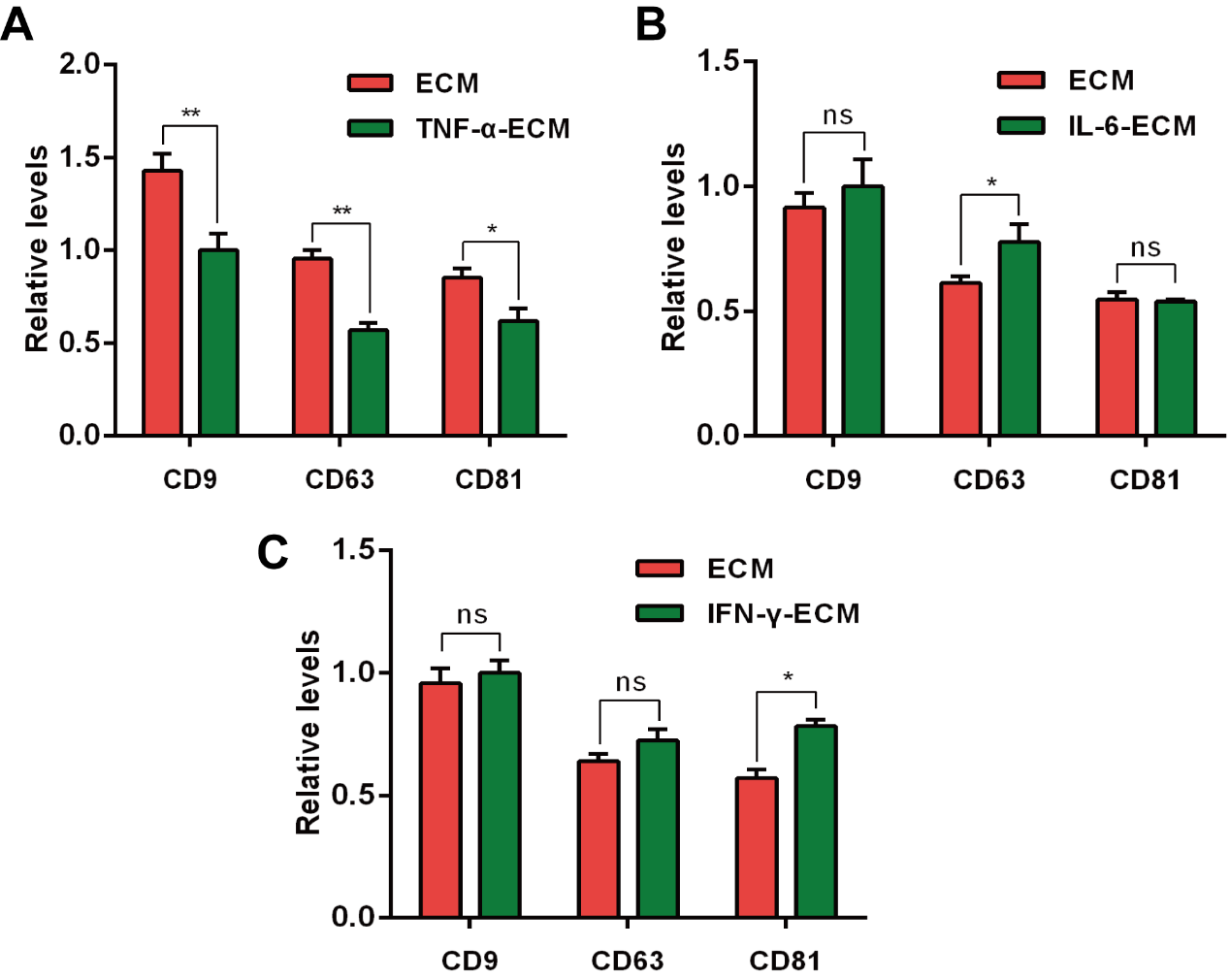


**Fig. S1:** Proteomic analysis reveals altered expression levels of CD9, CD63, and CD81. (A) TNF-α-ECM versus ECM. (B) IL-6-ECM versus ECM. (C) IFN-γ-ECM versus ECM. *p < 0.05, **p < 0.01. Data are presented as the mean ± SD (n = 3)





**Fig. S2:** Evaluation of the effects of engineered ECM on the proliferation of IL-1β-induced chondrocytes. *p < 0.05, **p < 0.01. Data are presented as the mean ± SD (n = 3)


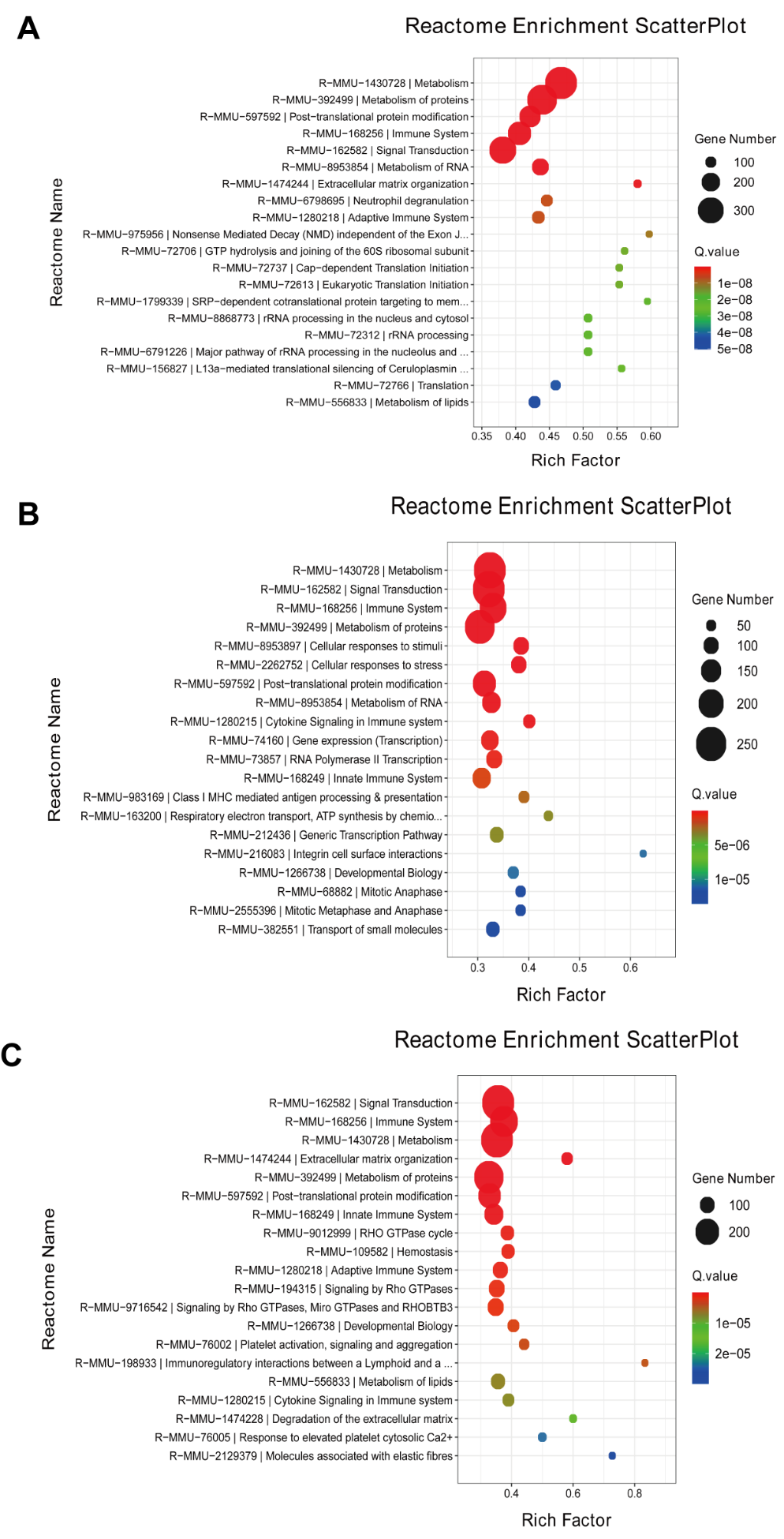


**Fig. S3:** Reactome enrichment analysis comparing differential protein enrichment between engineered and natural ECM. (A) TNF-α-ECM versus ECM. (B) IL-6-ECM versus ECM. (C) IFN-γ-ECM versus ECM.


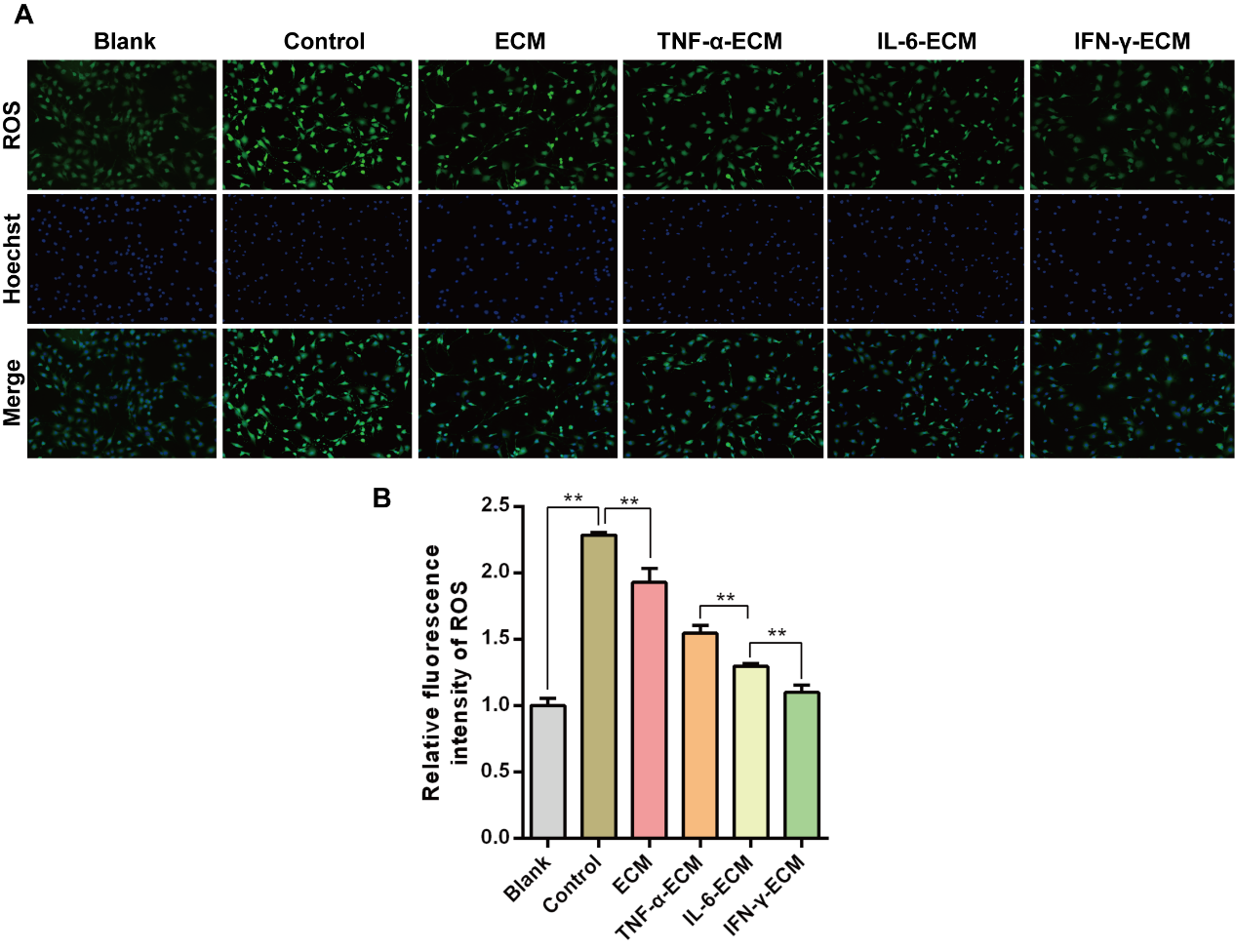


**Fig. S4:** Effects of engineered ECM on ROS levels in chondrocytes. *p < 0.05, **p < 0.01. Data are presented as the mean ± SD (n = 4)

**Table S1.** Primers used in real-time PCR.

| **Gene** | **Primer** | **Sequence** |
| --- | --- | --- |
| GAPDH | Forward | CAGTGGCAAAGTGGAGATTGTTG |
|  | Reverse | TCGCTCCTGGAAGATGGTGAT |
| Col II | Forward | CAGGGCTCCAATGATGTAGAGAT |
|  | Reverse | CCATGGGTGCGATGTCAATAATG |
| Aggrecan | Forward | CTGACGGACACTCTCTGCAATTT |
| Sox9  ADAMTS5  IL-1β  TNF-α  IL-6 | Reverse  Forward  Reverse  Forward  Reverse  Forward  Reverse  Forward  Reverse  Forward  Reverse | CCTCTCTCATGCCAGATCATCAC  CACGGAACAGACTCACATCTCTC  CCCTCTCGCTTCAGATCAACTTT  AGAACTGGATGTGACGGCATTAT  GGGATCCTCACAACGTCAGTATAA  CAGCACATCAACAAGAGCTTCAG  GAGGATGGGCTCTTCTTCAAAGA  CCAGACCCTCACACTCAGATCAT  AGAACCTGGGAGTAGACAAGGTA  TTAGCCACTCCTTCTGTGACTCC  ACCCCAATTTCCAATGCTCT |
| ITGA2 | Forward | ACAAGCCCGTGATCTTTCCTAAA |
|  | Reverse | GGTCACATTGCCTTGCTTGTTAA |
| ITGA5 | Forward | GCTGGACTGTGGTGAAGACAATA |
| ITGB1 | Reverse  Forward  Reverse | AATGTCAGGTTCAGTGCGTTCTT  GGAGATGGGAAACTTGGTGGTAT  CCCACTGCTGACTTAGGAATCAA |
